# Supplementary material for: The causal relationship between the human gut microbiota and pyogenic arthritis: a Mendelian randomization study
Source: Front Cell Infect Microbiol. 2024 Nov 26;14:1452480. doi: 10.3389/fcimb.2024.1452480 (PMC11629706; doi:10.3389/fcimb.2024.1452480)
Supplement: Supplementary file 1 [file DataSheet1.zip › mendelian test/ebi-a-GCST90027807.csv_forest.pdf]

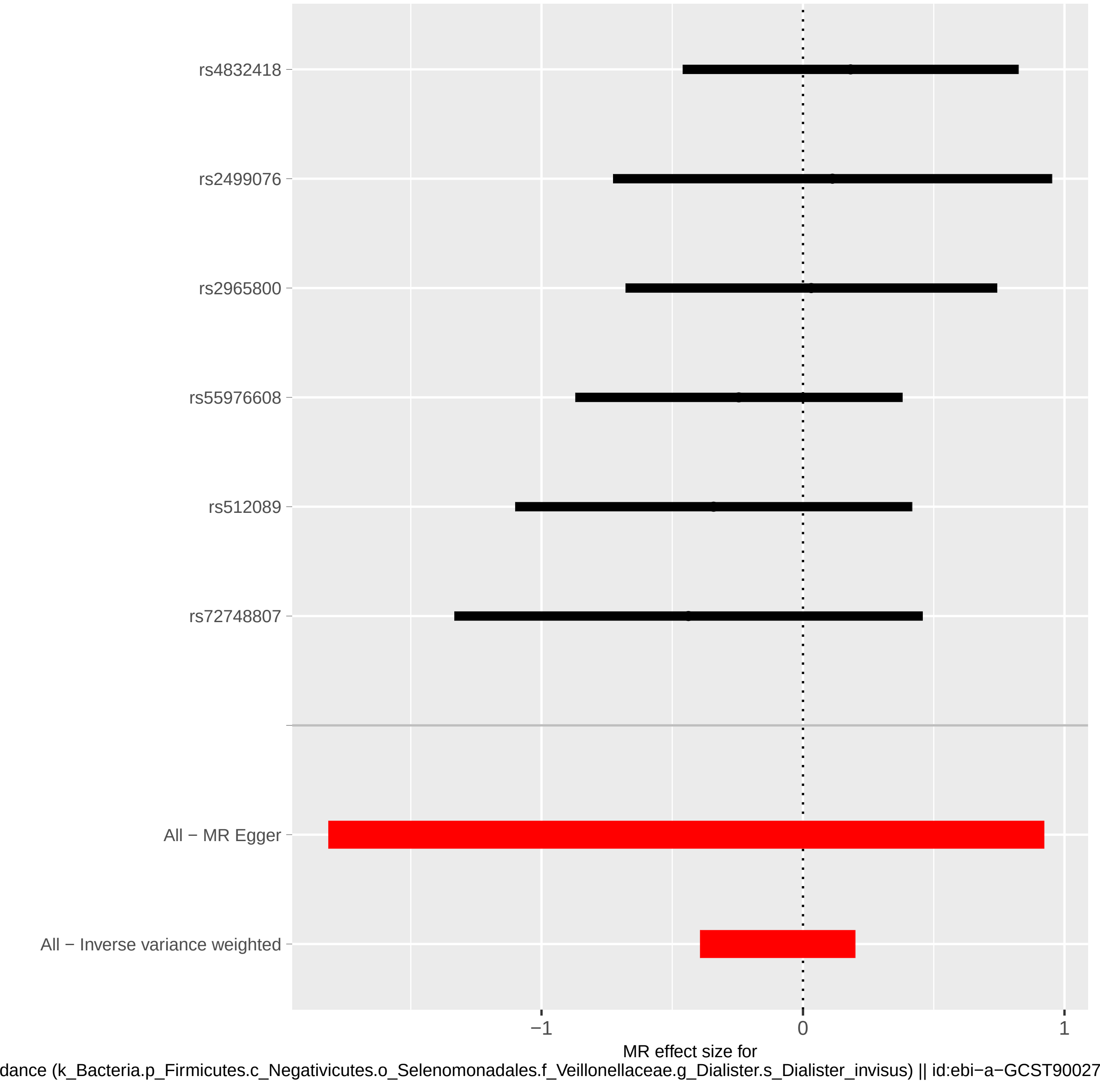
rs4832418
rs2499076
rs2965800
rs55976608
rs512089
rs72748807
All − MR Egger
All − Inverse variance weighted
−1
0
1
MR effect size for
dance (k_Bacteria.p_Firmicutes.c_Negativicutes.o_Selenomonadales.f_Veillonellaceae.g_Dialister.s_Dialister_invisus) || id:ebi−a−GCST90027
